# Analysis of 12 GWAS-Linked Loci With Parkinson’s Disease in the Chinese Han Population

**DOI:** 10.3389/fneur.2021.623913

**Published:** 2021-04-07

**Authors:** Liyuan Fan, Changhe Shi, Xinchao Hu, Zhongxian Zhang, Huimin Zheng, Haiyang Luo, Yu Fan, Shuo Zhang, Zhengwei Hu, Jing Yang, Chengyuan Mao, Yuming Xu

**Affiliations:** ^1^Department of Neurology, The First Affiliated Hospital of Zhengzhou University, Zhengzhou University, Zhengzhou, China; ^2^The Academy of Medical Sciences of Zhengzhou University, Zhengzhou University, Zhengzhou, China; ^3^Henan Key Laboratory of Cerebrovascular Diseases, The First Affiliated Hospital of Zhengzhou University, Zhengzhou University, Zhengzhou, China; ^4^Institute of Neuroscience, Zhengzhou University, Zhengzhou, China; ^5^Sino-British Research Centre for Molecular Oncology, National Centre for International Research in Cell and Gene Therapy, School of Basic Medical Sciences, Academy of Medical Sciences, Zhengzhou University, Zhengzhou, China

**Keywords:** Parkinson's disease, single nucleotide polymorphisms, Chinese population, *GBF1*, *C5orf24*, *GS1-124K5*·*11*

## Abstract

A recent large-scale European-originated genome-wide association study identified 38 novel independent risk signals in 37 loci for Parkinson's disease (PD). However, whether these new loci are associated with PD in Asian populations remains elusive. The present study aimed to explore the relationship between the 12 most relevant loci with larger absolute values for these new risk loci and PD in the Chinese Han population. We performed a case-control study including 527 PD patients and 435 healthy controls. In the allele model, it was found that rs10748818/*GBF1* was associated with PD in the Chinese Han population [*p* = 0.035, odds ratio (OR) 1.221, 95% confidence interval (CI) 1.014–1.472

## Introduction

Parkinson's disease (PD) is the second most common neurodegenerative disorder next to Alzheimer's disease, with a prevalence of 1.7% in the Chinese Han population aged ≥ 65 years (1). The majority of PD cases are sporadic with elusive etiology. Varying factors contribute to the development of PD, including environmental and genetic factors. Mounting evidence has revealed that the latter may provide significant clues to causes of PD (2).

Genome-wide association study (GWAS)-related loci, such as single nucleotide polymorphisms (SNPs) in SNCA, GBA, and LRRK2, are reported to be associated with PD (3). Pooling 17 datasets from PD GWAS available from European ancestry samples, a recent large-scale meta-analysis identified 38 novel independent risk signals in 37 loci for PD (4). However, whether these new loci are associated with PD in Asian populations remains elusive. Referring to the regression coefficient in the results of this meta-analysis, we selected the 12 most relevant loci with larger absolute values for exploration (The detailed information of 38 SNPs was shown in Supplementary Table 2). Consequently, a study including 527 PD patients and 435 healthy controls was performed to investigate the association between the 12 new loci and PD in the Chinese Han population.

## Materials and Methods

### Subjects

A total of 962 subjects of Han Chinese ethnicity were enrolled in the study, including 527 sporadic PD patients and 435 controls. The mean age and sex ratio (male/female) of the two groups were (PD patients: 62.34 ± 9.150 years, 300/227) and (healthy controls: 47.75 ± 10.856 years, 238/197), respectively. To minimize the effect of the familial PD, all patients recruited are sporadic cases. And the young-onset patients were excluded. The cases were defined using the United Kingdom Parkinson's Disease Society Brain Bank criteria. All subjects participating in the study signed written informed consent. This study was approved by the Ethics Committee of First Affiliated Hospital of Zhengzhou University.

### Genotyping and Data Analysis

Genomic DNA was extracted from peripheral blood collected from the patients and controls using the Blood Genome Extraction Kit (BioTeke Co, Beijing, China). SNPs were genotyped using improved multiple ligase detection reaction (iMLDR) technology (Geneskybiotech, Shanghai, China). All relevant specific polymerase chain reaction (PCR) primers and ligation primers were listed in Supplementary Table 1.

Statistical analysis was performed using IBM SPSS Statistics 26.0. The age difference was assessed using the *t* test. The Hardy-Weinberg equilibrium in genotype-frequency of controls was assessed using the χ2 test. Logistic regression analysis was used to calculate the risk analysis of each SNP in dominant, recessive models after adjusting for age and gender. Chi-squared tests were adopted to compare differences of sex ratio, genotype frequency, and allele frequency after age and gender-stratified analysis. Multiple tests were performed using the Bonferroni correction method. A 2-tailed *p* < 0.05 was considered statistically significant.

## Results

Frequencies of all 12 variants in the cases and controls met with Hardy-Weinberg equilibrium (*p* > 0.05, Table 1). In the allele model, the result showed that the rs10748818/*GBF1* variant exhibited significant difference between PD patients and the controls [*p* = 0.035, odds ratio (OR) 1.221, 95% confidence interval (CI) 1.014–1.472, Table 1]. A higher level of G allele was observed in the patients compared with the controls. In dominant and recessive models, rs10748818/*GBF1* was not associated with PD after sex and age adjustment *via* logistic regression [*p* = 0.275, (OR) 0.834, (CI) 0.601–1.155, Table 1]. In addition, age-stratified analysis showed rs11950533/*C5orf24* (genotype model: *p* =0.034, Table 2) and rs76949143/*GS1-124K5*·*11* (genotype model: *p* = 0.042, Table 2) were associated with early-onset PD (age < 50 years) and late-onset PD (age ≥ 50 years), respectively.

**Table 1:** Assessment of the relationship level of 12 novel loci with PD in the Chinese Han population.

**SNPs/candidate gene**	**HWE (*p*-value)**	**Association test**	**PD**	**Control**	***p***	**OR (95%CI)**	***p***^**a**^	**OR (95%CI)**^**a**^
rs10748818/*GBF1*	0.73	Genotypic (GG/GA/AA)	78/259/190	54/194/187	-	-		
		Dominant [(GG + GA)/AA]	337/190	248/187	0.028^*^	0.748(0.576–0.970)	0.275	0.834(0.601–1.155)
		Recessive [GG/(GA + AA)]	78/449	54/381	0.285	1.226(0.844–1.780)	0.857	1.030(0.745–1.424)
		Alleles(G/A)	415/639	302/508	0.035^*^	**1.221(1.014**–**1.472)**		
rs11950533/*C5orf24*	0.99	Genotypic (CC/CA/AA)	259/218/50	209/186/40	-	-		
		Dominant [(CC + CA)/AA]	477/50	395/40	0.877	1.035(0.669-1.602)	0.857	1.030 (0.745–1.424)
		Recessive [CC/(CA + AA)]	259/268	209/226	0.734	1.045(0.811–1.347)	0.416	0.875(0.634–1.208)
		Alleles(C/A)	736/318	604/266	0.848	1.019(0.839–1.239)		
rs34025766/*LCORL*	0.85	Genotypic (TT/AT/AA)	423/96/8	336/94/5	-	-		
		Dominant [(TT + AT)/AA]	519/8	430/5	0.622	0.754(0.245–2.323)	0.438	1.716(0.439–6.710)
		Recessive [TT/(AT + AA)]	423/104	336/99	0.253	1.198(0.879–1.634)	0.683	0.922(0.625–1.361)
		Alleles(T/A)	942/112	766/104	0.358	1.142(0.860–1.516)		
rs55961674/*KPNA1*	0.95	Genotypic (CC/CT/TT)	416/103/8	333/96/6	-	-		
		Dominant [(CC+CT)/TT]	519/8	429/6	0.858	0.907(0.312-2.635)	0.900	1.087(0.295–4.004)
		Recessive [CC/(CT+TT)]	416/111	333/102	0.375	1.148(0.846–1.557)	0.797	0.951(0.646-1.399)
		Alleles(C/T)	935/119	762/108	0.447	1.114(0.844–1.470)		
rs61169879/*BRIP1*	0.77	Genotypic (CC/CT/TT)	97/271/159	84/222/129	-	-		
		Dominant [(CC + CT)/TT]	368/159	306/129	0.862	0.976(0.739–1.288)	0.792	0.953(0.669–1.359)
		Recessive [CC/(CT + TT)]	97/430	84/351	0.721	0.943(0.681–1.304)	0.457	1.168(0.775–1.761)
		Alleles(C/T)	465/589	390/480	0.755	0.972(0.811–1.164)		
rs666463/*DNAH17*	0.81	Genotypic (AA/AT/TT)	498/29/0	409/26/0	-	-		
		Dominant [(AA + AT)/TT]	527/0	435/0	-	-	-	-
		Recessive [AA/(AT + TT)]	498/29	409/26	0.753	1.092(0.633–1.883)	0.337	1.421(0.693–2.912)
		Alleles(A/T)	1025/29	844/26	0.756	1.089(0.636–1.863)		
rs75859381/*RPS12*	0.60	Genotypic (TT/CT/CC)	467/59/1	395/40/0	-	-		
		Dominant [(TT + CT)/CC]	526/1	435/0	-	-	1.000	-
		Recessive [TT/(CT+CC)]	467/60	395/40	0.268	0.788(0.517–1.202)	0.939	0.980(0.578–1.660)
		Alleles(T/C)	993/61	830/40	0.244	0.785(0.521–1.181)		
rs76116224/*KCNS3*	0.99	Genotypic (AA/AT/TT)	523/4/0	429/6/0	-	-		
		Dominant [(AA+AT)/TT]	527/0	435/0	-	-	-	-
		Recessive [AA/(AT + TT)]	523/4	429/6	0.532^b^	0.541(0.149–1.957)	0.955	0.953(0.180–5.050)
		Alleles(A/T)	1,050/4	864/6	0.533^b^	1.823(0.513–6.480)		
rs76949143/*GS1-124K5·11*	0.24	Genotypic (TT/AT/AA)	385/135/7	333/90/12	-	-		
		Dominant [(TT + AT)/AA]	520/7	423/12	0.113	2.107(0.822–5.400)	0.438	0.624(0.189–2.054)
		Recessive [TT/(AT + AA)]	385/142	333/102	0.215	0.830(0.619–1.114)	0.688	1.080(0.740–1.576)
		Alleles(T/A)	905/149	756/114	0.511	0.916(0.705–1.191)		
rs77351827/*CRLS1*	1.00	Genotypic (CC)	527	435	-	-		
		-	-	-	-	-	-	-
		-	-	-	-	-	-	-
		Alleles(C)	1,054	870	-	-		
rs7938782/*RNF141*	0.36	Genotypic (AA/GA/GG)	373/143/11	303/115/17	-	-		
		Dominant [(AA + GA)/GG]	516/11	418/17	0.095	1.908(0.884–4.117)	0.120	0.469(0.181–1.217)
		Recessive [AA/(GA + GG)]	373/154	303/132	0.705	1.055(0.799–1.393)	0.951	1.011(0.710–1.440)
		Alleles(A/G)	889/165	721/149	0.341	0.889(0.697–1.133)		
rs850738/*FAM171A2*	0.68	Genotypic (AA/GA/GG)	184/248/95	144/220/71	-	-		
		Dominant [(AA + GA)/GG]	432/95	364/71	0.486	0.887(0.633–1.243)	0.186	1.337(0.869–2.056)
		Recessive [AA/(GA + GG)]	184/343	144/291	0.555	1.084(0.829–1.418)	0.775	1.051(0.748–1.477)
		Alleles(A/G)	616/438	508/362	0.981	1.002(0.835–1.202)		

* *A two-tailed p < 0.05 was considered significant*.

a *Adjusted age and sex by logistic regression*.

b *Continuous correction for Chi-square test when at least one cell has an expected value of < 5*.

*PD, Parkinson's disease; SNPs, single nucleotide polymorphisms; HWE, Hardy-Weinberg equilibrium; CI, confidence interval; OR, odds ratio. The bold means the P value < 0.05*.

**Table 2:** Age-stratified analysis and sex-stratified analysis of 12 loci.

**SNPs (candidate gene)**	**Genotype, allele**	**Age onset < 50 years**				**Age onset** **≥** **50 years**				**Male**				**Female**			
		**PD**	**Control**	***p*_**1**_**	**OR (95%CI)_**1**_**	**PD**	**Control**	***p*_**2**_**	**OR (95%CI)_**2**_**	**PD**	**Control**	***p*_**3**_**	**OR (95%CI)_**3**_**	**PD**	**Control**	***p*_**4**_**	**OR (95%CI)_**4**_**
rs10748818	GG	7	28	0.370		71	26	0.132		44	27	0.126		34	27	0.524	
(*GBF1*)	GA	16	113			243	81			146	107			131	87		
	AA	23	106			167	81			110	104			80	83		
	G	30	169	0.766	0.931 (0.579–1.495)	385	133	0.117	1.219(0.952–1.562)	234	161	0.080	1.251(0.973–1.608)	181	141	0.222	1.190(0.900–1.572)
	A	62	325			577	243			366	315			273	253		
**rs11950533**	CC	24	119	0.034^*^		235	90	0.783		146	108	0.704		113	101	0.954	
**(*****C5orf24*****)**	CA	17	103			201	83			123	106			95	80		
	AA	5	25			45	15			31	24			19	16		
	C	65	341	0.757	1.080 (0.663–1.759)	671	263	0.944	0.991(0.764–1.285)	415	322	0.594	1.073(0.828–1.390)	321	282	0.781	0.959(0.712–1.291)
	A	27	153			291	113			185	154			133	112		
rs34025766	TT	38	188	-		385	148	0.418		240	190	-		183	146	-	
(*LCORL*)	AT	8	55			88	39			55	46			41	48		
	AA	0	4			8	1			5	2			3	3		
	T	84	431	0.274	1.535 (0.709–3.321)	858	335	0.961	1.010(0.689–1.481)	535	426	0.862	0.966(0.654–1.427)	407	340	0.133	1.375(0.907–2.086)
	A	8	63			104	41			65	50			47	54		
rs55961674	CC	36	190	-		380	143	0.568		239	185	-		177	148	-	
(*KPNA1*)	CT	8	55			95	41			57	49			46	47		
	TT	2	2			6	4			4	4			4	2		
	C	80	435	0.767	0.904 (0.465–1.758)	855	327	0.328	1.197(0.834–1.718)	535	419	0.558	1.120(0.767–1.634)	400	343	0.643	1.101(0.732–1.658)
	T	12	59			107	49			65	57			54	51		
rs61169879	CC	9	45	0.673		88	39	0.439		55	40	0.544		42	44	0.351	
(*BRIP1*)	CT	22	135			249	87			147	128			124	94		
	TT	15	67			144	62			98	70			61	59		
	C	40	225	0.714	0.920(0.587–1.440)	425	165	0.922	1.012(0.796–1.287)	257	208	0.776	0.965(0.757–1.231)	208	182	0.912	0.985(0.751–1.291)
	T	52	269			537	211			343	268			246	212		
rs666463	AA	42	229	0.978^a^		456	180	0.613		284	222	0.499		214	187	0.768	
(*DNAH17*)	AT	4	18			25	8			16	16			13	10		
	-	-	-			-	-			-	-			-	-		
	A	88	476	0.978^a^	0.832(0.275–2.517)	937	368	0.617	0.815(0.364–1.823)	584	460	0.505	1.270(0.628–2.566)	441	384	0.771	0.883(0.383–2.037)
	T	4	18			25	8			16	16			13	10		
rs75859381	TT	41	226	0.813^a^		426	169	-		267	219	0.240		200	176	-	
(*RPS12*)	CT	5	21			54	19			33	19			26	21		
	CC	0	0			1	0			0	0			1	0		
	T	87	473	0.818^a^	0.773(0.284–2.104)	906	357	0.583	0.861(0.504–1.470)	567	457	0.252	0.714(0.401–1.273)	426	373	0.602	0.857(0.478–1.534)
	C	5	21			56	19			33	19			28	21		
rs76116224	AA	45	242	1.000^b^		478	187	1.000^a^		296	236	0.899^a^		227	193	0.098^a^	
(*KCNS3*)	AT	1	5			3	1			4	2			0	4		
	-	-	-			-	-			-	-			-	-		
	A	91	489	1.000^b^	0.930(0.107–8.057)	959	375	1.000^a^	0.571(0.153–2.133)	596	474	0.899^a^	0.629(0.115–3.447)	454	390	0.099^a^	0.462(0.430–0.497)
	T	1	5			3	1			4	2			0	4		
**rs76949143**	TT	38	191	-		347	142	0.042^*^		217	184	0.241		168	149	-	
**(*****GS1-124K5·11*****)**	AT	7	51			128	39			78	48			57	42		
	AA	1	5			6	7			5	6			2	6		
	A	9	61			140	53			88	60			61	54		
rs77351827	CC	46	247	-		481	188	-	-	300	238	-	-	227	197	-	-
(*CRLS1*)	C	92	494	-	-	962	376	-	-	600	476	-	-	454	394	-	-
rs7938782	AA	33	176	0.969		340	127	0.085		210	162	0.327		163	141	0.693	
(*RNF141*)	GA	12	64			131	51			84	66			59	49		
	GG	1	7			10	10			6	10			5	7		
	A	78	416	0.890	1.045(0.563–1.938)	811	305	0.159	1.250(0.916–1.707)	504	390	0.433	0.880(0.638–1.212)	385	331	0.751	1.062(0.732–1.540)
	G	14	78			151	71			96	86			69	63		
rs850738	AA	15	74	0.843		169	70	0.300		102	82	0.345		82	62	0.587	
(*FAM171A2*)	GA	24	127			224	93			140	121			108	99		
	GG	7	46			88	25			58	35			37	36		
	A	54	275	0.591	1.132(0.721–1.777)	562	233	0.235	0.862(0.675–1.101)	344	285	0.401	0.901(0.705–1.150)	272	223	0.329	1.146(0.872–1.507)
	G	38	219			400	143			256	191			182	171		

* *A two-tailed p < 0.05 was considered significant*.

a *Continuous correction for Chi-square test when at least one cell has an expected value of less than 5*.

b *Adjusted by Fisher's exact test when at least one cell has an expected value of < 1*.

*PD, Parkinson's disease; SNPs, single nucleotide polymorphisms; CI, confidence interval; OR, odds ratio. The bold means the P value < 0.05*.

In contrast, no statistical difference in genotype or allele frequency was detected between PD patients and the controls in the remaining nine loci (rs34025766/*LCORL*, rs55961674/*KPNA1*, rs61169879/*BRIP1*, rs666463/*DNAH17*, rs75859381/*RPS12*, rs76116224/*KCNS3*, rs77351827/*CRLS1*, rs7938782/*RNF141*, and rs850738/*FAM171A2*, Tables 1, 2), neither between groups of the same sex or the same age. All detailed information on the relationship level of 12 loci with PD is shown in Tables 1, 2.

## Discussion

Given the effects of ethnic heterogeneity, our present study investigated the 12 new identified PD-associated variants in a Han Chinese population. We demonstrated that rs10748818/*GBF1* exhibited a difference between PD patients and the controls in the allele mode. After age-stratified analysis, rs11950533/*C5orf24* and rs76949143/*GS1-124K5*·*11* were associated with early-onset PD and late-onset PD, respectively. To the best of our knowledge, our study is the first to show the association of SNPs in *GBF1, C5orf24*, and *GS1-124K5*·*11* genes. No statistical difference in genotype or allele frequency was detected between PD patients and the controls in the remaining nine loci. Our study, however, failed to replicate the association of the reported SNPs with PD by Nalls et al. in the European population, which may partially be due to the genetic heterogeneity caused by ethnic and geographical differences (detailed 38 loci information of the GWAS results by Nalls et al. are shown in Supplementary Table 2). Additionally, the interaction between environmental and genetic factors may influence gene expression.

The first Han Chinese GWAS by Foo JN analyzed a total of 22,729 subjects (5,125 PD cases and 17,604 controls) from Singapore, Hong Kong, Malaysia, Korea, mainland China, and Taiwan and replicated associations at *SNCA, LRRK2, MCCC1*, and 14 other European PD loci but did not identify Asian-specific loci with large effects on PD risk (5). A two-stage meta-analysis of GWAS identified 17 new loci, which were associated with the risk of PD in the European population (26,000 PD patients and 403,000 healthy controls). However, the following study did not find any association between the five most commonly identified candidate variants in the European population with PD in the Chinese population (506 PD patients, 496 MSA patients, and 894 age- and sex-matched healthy controls) (6). Recently, we reported that rs34043159 of *IL1R2* and rs4073221 of *SATB1* were associated with PD in Chinese Han people (492 PD patients and 524 healthy controls). Further subgroup analysis showed that both rs34043159 of *IL1R2* and rs4073221 of *SATB1* were associated with late-onset PD. rs34043159 of *IL1R2* was associated with PD in female patients, while rs4073221 of *SATB1* was associated with PD in both male and female patients (7). The two loci were suggested to be involved in the pathogenesis of PD. But there are still more genetic factors to be identified. Here, we identified another three loci, which were associated with the increased risk of PD.

*GBF1*, also named *ARF1GEF*, encodes a member of the Sec7 domain family, which is a guanine nucleotide exchange factor and activates small GTPases of the Arf family. It is involved in regulating the recruitment of proteins to membranes and has been reported to play an essential role in the regulation of the spatial organization and function of mitochondria in a microtubule-dependent manner (8). Numerous studies have implicated that mitochondrial and apoptosis dysfunction are both strongly linked with PD pathogenesis (9). GBF1 localizes at the early Golgi (10) and also links to lipid droplet metabolism (11), plasma membrane signaling, and organelle transport along microtubules with its substrate Arf1. Furthermore, it is involved in the regulation of Golgi fragmentation and is essential for Golgi disassembly and subsequent mitosis entry (12). The fragmentation of the Golgi apparatus is an essential process in the development of apoptosis, which may be related to PD susceptibility. These studies indicated the association of *GBF1* with PD.

The *C5orf24* is chromosome 5 open reading frame 24, and it has been shown that its DNA methylation level is related to negative affect scores in drug addicts (13). A study identified C5orf24 was upregulated in patients with posttraumatic stress disorder (PTSD) and high intrusion symptoms at baseline and downregulated in participants following treatment (14). However, further investigations are needed to explore the roles of *C5orf24* genes played in pathophysiologic pathways of PD.

*GS1-124K5*·*11* is the RAB guanine nucleotide exchange factor 1 pseudogene. The related functional gene of *GS1-124K5*·*11* is *RAB guanine nucleotide exchange factor 1* (*RABGEF1*), which is the upstream factor of the endosomal Rab GTPase cascade. Mutations in *Parkin* are the second-most-common known cause of PD, and *Parkin* plays a critical role in mitophagy through ubiquitination of mitochondria. *RABGEF1* is recruited to damaged mitochondria *via* ubiquitin binding downstream of Parkin in mammalian cultured cells and promotes autophagy of damaged mitochondria (15). Overexpression of A53T-Alpha-Synuclein upregulated the expression of RABGEF1 in the mouse midbrain/brainstem (16). However, the role of *GS1-124K5*·*11* in the pathogenesis of PD needs to be further explored.

There are several limitations in the current study, such as the relatively small sample size. Noteworthy, the molecular mechanisms between rs10748818/*GBF1*, rs11950533/*C5orf24*, rs76949143/*GS1-124K5*·*11*, and PD are still unclear, so more functional experiments should be designed to explore the pathogenesis.

In conclusion, our study demonstrated that the variants of *GBF1, C5orf24*, and *GS1-124K5*·*11* are associated with PD in the Han Chinese population. It remains to be determined whether geographic or environmental factors are involved in the genetic consequences of these loci. Further genetic analysis and function studies are needed to understand the role of these variants in the pathogenesis of PD.

## Data Availability Statement

The original contributions presented in the study are included in the article/Supplementary Materials, further inquiries can be directed to the corresponding author/s.

## Ethics Statement

The studies involving human participants were reviewed and approved by the Ethics Committee of First Affiliated Hospital of Zhengzhou University. The patients/participants provided their written informed consent to participate in this study.

## Author Contributions

LF: data curation, formal analysis, and writing-original draft. CS: resources and funding acquisition. XH and HZ: formal analysis. CM: conceptualization and funding acquisition. YX: funding acquisition and supervision. ZZ: methodology. YF: data curation. HL, SZ, and ZH: writing—review & editing. JY: supervision. All authors: contributed to the study's conception and design.

## Conflict of Interest

The authors declare that the research was conducted in the absence of any commercial or financial relationships that could be construed as a potential conflict of interest.

## Abbreviations

PD: parkinson's disease
OR: odds ratio
CI: 95% confidence interval
GWAS: genome-wide association study
SNPs: single nucleotide polymorphisms
PTSD: post-traumatic stress disorder
HWE: Hardy-Weinberg equilibrium
RABGEF1: RAB guanine nucleotide exchange factor 1.
