# Supplementary material for: Analysis of 12 GWAS-Linked Loci With Parkinson’s Disease in the Chinese Han Population
Source: Front Neurol. 2021 Apr 7;12:623913. doi: 10.3389/fneur.2021.623913 (PMC8058430; doi:10.3389/fneur.2021.623913)
Supplement: Supplementary file 1 [file Data_Sheet_1.docx]

**Supplementary Table 1.** PCR primers and Ligation primers.

| SNPs | Category 1 | PCR primers | Category 2 | Ligation primers |
| --- | --- | --- | --- | --- |
| rs10748818 | FP | CGCCCAGCCCTAGATGTTTCTT | RA | TGTTCGTGGGCCGGATTAGTGGTGGAGGAGTGAAAGGAAGACTAACTGTT |
|  | RP | GAATGTACAACCACGCTCTCTTCTG | RG | TCTCTCGGGTCAATTCGTCCTTGGTGGAGGAGTGAAAGGAAGACTAACTGTC |
|  | - | - | RP | CTCTGTATATTCTTTTTTACCTTTTGAATTTTGTG |
| rs11950533 | FP | TGGAATTCCCATCTCCTCCAGAT | FA | TACGGTTATTCGGGCTCCTGTGCAGAACAGCCCTTCTCACCTCA |
|  | RP | TCCCATAACATGAAAAACAGCATTAGTAG | FC | TTCCGCGTTCGGACTGATATGCAGAACAGCCCTTCTCACCTCC |
|  | - | - | FP | CTTCTTTGTGGGAGCTATTCTGCCTTTTTTTTTTTTTTTTTTTTTTTTTTTT |
|  | - | - | FT | TCTCTCGGGTCAATTCGTCCTTGCAGAACAGCCCTTCTCACCTCT |
| rs34025766 | FP | GCCACATTTTGAAAGTGAGCAAGA | FA | TCTCTCGGGTCAATTCGTCCTTCTTTAGAAGCTACATACTGTATGATTCCAACTAGAA |
|  | RP | CTTCCACTTCCCCAAACCTGTG | FP | GACATTCTAAAAAAAACAAAACTTTAGAGACCA |
|  | - | - | FT | TGTTCGTGGGCCGGATTAGTCTTTAGAAGCTACATACTGTATGATTCCAACTAGAT |
| rs55961674 | FP | ATTTGCTTGATTTAGCCATTCCACA | RC | TCTCTCGGGTCAATTCGTCCTTCCTCAGCCTCCCAAGTAGCAGG |
|  | RP | CAGCACCATTTCCTCTCTTTGGAC | RP | CATTAGAGGCACGTGCCACCTTTTT |
|  | - | - | RT | TGTTCGTGGGCCGGATTAGTCCTCAGCCTCCCAAGTAGCAGA |
| rs61169879 | FP | CAAAAGGAGAAATCAATCAAAGCATTAG | FC | TCTCTCGGGTCAATTCGTCCTTTCAACCAAGGGCACAGTAGATGAGC |
|  | RP | CTGTTGAAGATGTTTATGGCTGAAATC | FP | TTATGGTCAAGCGGTTTAGACTYAATTT |
|  | - | - | FT | TGTTCGTGGGCCGGATTAGTTCAACCAAGGGCACAGTAGATGAGT |
| rs666463 | FP | GGCACCTGAGGAAGGATGACAG | RA | TTCCGCGTTCGGACTGATATCGAGAAGGAYRCAGCACCCAT |
|  | RP | GCCTGGTGACCCTGAAGGTAAA | RP | GGCTGGGTTCCCACGCCCTTTTTTTT |
|  | - | - | RT | TACGGTTATTCGGGCTCCTGTCGAGAAGGAYRCAGCACCCAA |
| rs75859381 | FP | CAGATGTGCAGCCCTGTTTTCA | FC | TTCCGCGTTCGGACTGATATGCCCTGTTTTCAAGGGTTCTTAAGAC |
|  | RP | TTTCTCAGCCCATGTGGGATTT | FP | CTTTTCTGTAATATAGATTACATCCYRATCATG |
|  | - | - | FT | TACGGTTATTCGGGCTCCTGTGCCCTGTTTTCAAGGGTTCTTAAGAT |
| rs76116224 | FP | GCAGAGGCAGATGATGGTTTGA | FA | TTCCGCGTTCGGACTGATATAGCATGGTGTTGATATTTGATCTATTTCGA |
|  | RP | CCAGGGTAAGCGAGGGAGAGAT | FP | GTTGACTTTCTAACTCTCTGTAACTCCTTTGC |
|  | - | - | FT | TACGGTTATTCGGGCTCCTGTAGCATGGTGTTGATATTTGATCTATTTCGT |
| rs76949143 | FP | GACAGACCAACAACTGGGGCTA | FA | TCTCTCGGGTCAATTCGTCCTTCAGAGCGAGACTCTGTCTCAAAAAATAACTA |
|  | RP | CTGAGAGTTGGCACTGGCTTG | FP | AATWAATTAATTAAATAAAATAAATACGTAACTAAACAATT |
|  | - | - | FT | TGTTCGTGGGCCGGATTAGTCAGAGCGAGACTCTGTCTCAAAAAATAACTT |
| rs77351827 | FP | GCTAGAGGCCAATGAAGCCAGT | FC | TCTCTCGGGTCAATTCGTCCTTCAACATGATGTGCTGAATATTTTAAGCCAAC |
|  | RP | GTCACCTATAGCCTTGGGGAATGT | FP | AATTGAGACCTACTGCTCAGAAAAAAAAG |
|  | - | - | FT | TGTTCGTGGGCCGGATTAGTCAACATGATGTGCTGAATATTTTAAGCCGAT |
| rs7938782 | FP | CGCACCATCCAGAAGTGGAAAT | FA | TACGGTTATTCGGGCTCCTGTCCCTCTCATTGGAAGTATGGAAGTGTGTA |
|  | RP | TCCACTTCCCACCCAGGTAAAA | FG | TTCCGCGTTCGGACTGATATCCCTCTCATTGGAAGTATGGAAGTGTGTG |
|  | - | - | FP | GGTAAAGATTAGAAGTACATCTAATAAGTGTTACAGGG |
| rs850738 | FP | ATAAAATGTTGCGATCCAGATCCTC | FA | TGTTCGTGGGCCGGATTAGTTGAGGCTGAGGCAGGAGAAGCA |
|  | RP | TCTTGATGCCCAGACAGGAGTG | FG | TCTCTCGGGTCAATTCGTCCTTTGAGGCTGAGGCAGGAGAAACG |
|  | - | - | FP | CTTGAAACTGGGAGGGGGAGTTTTTTTT |

Key: SNP (single nucleotide polymorphism); PCR (polymerase chain reaction); FP (Forward primer), RP (Reverse primer) in Category 1 and FX (Forward base), RX (Reverse base) in Category 2.

**Supplementary Table 2.** The detailed information of 38 SNPs reported by Nalls et al.

| SNPs | Chromosome | Base pair position | Nearest gene | Effect allele | Other allele | OR (95% CI) | Regression coefficient (β) |
| --- | --- | --- | --- | --- | --- | --- | --- |
| rs10748818 | 10 | 104015279 | *GBF1* | A | G | 0.92 (0.90–0.95) | –0.079 |
| rs11950533 | 5 | 134199105 | *C5orf24* | A | C | 0.91 (0.88–0.94) | –0.092 |
| rs34025766 | 4 | 17968811 | *LCORL* | A | T | 0.92 (0.90–0.94) | –0.084 |
| rs55961674 | 3 | 122196892 | *KPNA1* | T | C | 1.09 (1.06–1.12) | 0.086 |
| rs61169879 | 17 | 59917366 | *BRIP1* | T | C | 1.09 (1.06–1.11) | 0.082 |
| rs666463 | 17 | 76425480 | *DNAH17* | A | T | 1.08 (1.05–1.11) | 0.076 |
| rs75859381 | 6 | 133210361 | *RPS12* | T | C | 0.80 (0.75–0.86) | –0.221 |
| rs76116224 | 2 | 18147848 | *KCNS3* | A | T | 1.12 (1.08–1.16) | 0.110 |
| rs76949143 | 7 | 66009851 | *GS1-124K5.11* | A | T | 0.87 (0.82–0.91) | –0.143 |
| rs77351827 | 20 | 6006041 | *CRLS1* | T | C | 1.08 (1.05–1.11) | 0.080 |
| rs7938782 | 11 | 10558777 | *RNF141* | A | G | 1.09 (1.06–1.12) | 0.087 |
| rs850738 | 17 | 42434630 | *FAM171A2* | A | G | 0.93 (0.91–0.95) | –0.071 |
| rs6658353 | 1 | 161469054 | *FCGR2A* | C | G | 1·07 (1·05–1·09) | 0·065 |
| rs11578699 | 1 | 171719769 | *VAMP4* | T | C | 0·93 (0·91–0·95) | –0·070 |
| rs2042477 | 2 | 96000943 | *KCNIP3* | A | T | 0·94 (0·92–0·96) | –0·066 |
| rs6808178 | 3 | 28705690 | *LINC00693* | T | C | 1·07 (1·05–1·09) | 0·066 |
| rs11707416 | 3 | 151108965 | *MED12L* | A | T | 0·94 (0·92–0·96) | –0·063 |
| rs1450522 | 3 | 161077630 | *SPTSSB* | A | G | 0·94 (0·92–0·96) | –0·062 |
| rs62333164 | 4 | 170583157 | *CLCN3* | A | G | 0·94 (0·92–0·96) | –0·064 |
| rs26431 | 5 | 102365794 | *PAM* | C | G | 1·06 (1·04–1·09) | 0·062 |
| rs9261484 | 6 | 30108683 | *TRIM40* | T | C | 0·94 (0·92–0·96) | –0·064 |
| rs12528068 | 6 | 72487762 | *RIMS1* | T | C | 1·07 (1·05–1·09) | 0·066 |
| rs997368 | 6 | 112243291 | *FYN* | A | G | 1·07 (1·05–1·10) | 0·071 |
| rs2086641 | 8 | 130901909 | *FAM49B* | T | C | 0·94 (0·92–0·96) | –0·061 |
| rs6476434 | 9 | 34046391 | *UBAP2* | T | C | 0·94 (0·92–0·96) | –0·062 |
| rs7134559 | 12 | 46419086 | *SCAF11* | T | C | 0·95 (0·93–0·97) | –0·054 |
| rs11610045 | 12 | 133063768 | *FBRSL1* | A | G | 1·06 (1·04–1·08) | 0·060 |
| rs9568188 | 13 | 49927732 | *CAB39L* | T | C | 1·06 (1·04–1·09) | 0·062 |
| rs4771268 | 13 | 97865021 | *MBNL2* | T | C | 1·07 (1·05–1·09) | 0·068 |
| rs12147950 | 14 | 37989270 | *MIPOL1* | T | C | 0·95 (0·93–0·97) | –0·053 |
| rs3742785 | 14 | 75373034 | *RPS6KL1* | A | C | 1·07 (1·05–1·10) | 0·071 |
| rs2904880 | 16 | 28944396 | *CD19* | C | G | 0·94 (0·92–0·96) | –0·065 |
| rs6500328 | 16 | 50736656 | *NOD2* | A | G | 1·06 (1·04–1·08) | 0·059 |
| rs12600861 | 17 | 7355621 | *CHRNB1* | A | C | 0·95 (0·93–0·96) | –0·057 |
| rs2269906 | 17 | 42294337 | *UBTF* | A | C | 1·07 (1·04–1·09) | 0·063 |
| rs1941685 | 18 | 31304318 | *ASXL3* | T | G | 1·05 (1·04–1·07) | 0·053 |
| rs8087969 | 18 | 48683589 | *MEX3C* | T | G | 0·94 (0·93–0·96) | –0·058 |
| rs2248244 | 21 | 38852361 | *DYRK1A* | A | G | 1·07 (1·05–1·10) | 0·071 |

Key: SNPs, single nucleotide polymorphisms; OR, odds ratio; CI, confidence interval.
